# Lnc13728 facilitates human mesenchymal stem cell adipogenic differentiation via positive regulation of ZBED3 and downregulation of the WNT/β-catenin pathway

**DOI:** 10.1186/s13287-021-02250-8

**Published:** 2021-03-12

**Authors:** Haoying Xu, Yanlei Yang, Linyuan Fan, Luchan Deng, Junfen Fan, Di Li, Hongling Li, Robert Chunhua Zhao

**Affiliations:** Institute of Basic Medical Sciences Chinese Academy of Medical Sciences, School of Basic Medicine Peking Union Medical College, Center of Excellence in Tissue Engineering Chinese Academy of Medical Sciences, Beijing Key Laboratory (No. BZO381), Beijing, 100005 China

**Keywords:** Long noncoding RNA 13728, Adipogenic differentiation, hADSCs, ZBED3, WNT/β-catenin signal pathway

## Abstract

**Background:**

Obesity has received increasing attention because of its widespread worldwide occurrence and many threats to health. Human adipose-derived mesenchymal stem cells (hADSCs) are a critical source of adipocytes. Long noncoding RNAs (lncRNAs) play pivotal roles in cell fate determination and differentiation. The objective of the present study was to identify and investigate the function and regulatory mechanism of lncRNAs on adipogenic differentiation of hADSCs.

**Methods:**

We used lncRNA arrays to identify the prominent differentially expressed lncRNAs before and after hADSC adipogenic differentiation and verified their biological function through antisense oligonucleotide knockdown or lentivirus overexpression. The adipogenic differentiation of hADSCs was assessed by oil red O staining as well as the mRNA and protein levels of adipogenic marker genes through qRT-PCR and western blot. Bioinformatic tool LncPro and immunofluorescence was performed to uncover the interaction between lnc13728 and ZBED3. WNT/β-catenin signaling pathway was evaluated by western blot and immunofluorescence.

**Results:**

The lncRNA arrays showed that lnc13728 expression was significantly upregulated after hADSC adipogenic differentiation and was correlated positively with the expression of the adipogenesis-related genes in human adipose tissue. Lnc13728 knockdown in hADSCs suppressed the expression of the adipogenesis-related genes at both mRNA and protein level and weakened lipid droplet production. Accordingly, lnc13728 overexpression enhanced hADSC adipogenic differentiation. Beyond that, lnc13728 co-localized with ZBED3 in the cytoplasm and regulated its expression positively. Downregulating *ZBED3* had a negative effect on adipogenic differentiation, while the expression of WNT/β-catenin signaling pathway-related proteins was upregulated.

**Conclusions:**

Lnc13728 promotes hADSC adipogenic differentiation possibly by positively regulating the expression of ZBED3 which plays a role in inhibiting the WNT/β-catenin pathway.

**Supplementary Information:**

The online version contains supplementary material available at 10.1186/s13287-021-02250-8.

## Background

As societies develop and lifestyles change, obesity has become a significant factor in threats to human physical and mental health. Obesity is not only the pathogenesis of the common type 2 diabetes mellitus (T2DM), promoting the development of diabetic complications, but is also the most important culprit in insulin resistance [1, 2]. Obesity indirectly increases the risk of coronary artery disease through dyslipidemia, hypertension, glucose intolerance, and endothelial dysfunction [3] and is also often accompanied by low-grade chronic inflammation, increased inflammatory factors, and other immunological diseases [4, 5].

In essence, obesity is caused by an abnormal increase in the number and volume of adipocytes, which are mainly derived from mesenchymal stem cells (MSCs) in vivo. Adipocytes play a major role in the pathogenesis of obesity and insulin resistance [1]. First, they act as “warehouses” for storing free fatty acid, accumulating lipids in a non-toxic manner [6]. When the storage capacity of adipocytes is exceeded, excessive lipid accumulation, lipotoxicity, and insulin resistance will occur, leading to lipodystrophy and obesity. Second, adipocytes secrete a variety of biologically active peptides (adipokines) to regulate insulin sensitivity. The obesity epidemic has led to scientists becoming interested in the development of adipose tissue and the stem/progenitor cells from which it originates [7, 8].

The differentiation of MSCs into adipocytes is a complicated process; the cascade of many transcription factors produces mature adipocytes through coordinated regulation [9]. This process starts with the transient expression of C/EBPβ and C/EBPδ to activate C/EBPα and PPARγ, which in turn promote the expression of adipocyte terminal differentiation-related adipogenic genes (e.g., *LPL* and *AP2*). To date, the regulation of adipogenesis involves many signaling pathways, such as TGFβ/BMPs/SMADs, WNT/β-catenin, Notch, JAK/STAT, MAPK, PI3K/Akt, and Hedgehog [10–14]. Identifying the important functional regulators in these biological processes is of great value for improving understanding of adipocyte development and expanding the therapeutic catalogue to intervene in obesity and other adipogenic differentiation-related diseases.

Long noncoding RNAs (lncRNAs) are > 200-bp transcripts that do not encode proteins. LncRNAs can regulate the expression of genes, proteins, or RNAs in a variety of ways in the cytoplasm and nucleus [15]. An increasing number of studies have found that lncRNAs are widely involved in regulating many cellular events, including stem cell pluripotency [16] and cell differentiation [17]. The impact of lncRNAs on important processes in the occurrence and development of obesity such as lipid metabolism and adipocyte differentiation has gradually been discovered. The lncRNA Sra1 is one of the first functional lncRNAs found in adipocytes. Sra1 knockout in mice can lead to insensitivity to high-fat diet (HFD)-induced obesity and glucose intolerance [18]. LncRNA-Adi was identified promoting adipogenesis of adipose tissue-derived stromal cells by interacting with miR-449a and increasing CDK6 translation to activate the pRb-E2F1 pathway [19]. PU.1 AS lncRNA facilitates adipogenesis through binding to PU.1 mRNA to form mRNA/AS lncRNA duplex and preventing PU.1 mRNA translation in preadipocytes [20]. Lnc-U90926 was demonstrated having an inhibitory role in adipogenesis in 3T3-L1 preadipocytes through reducing the transcription of PPARγ [21]. Most of the above studies of lncRNAs were carried out in mice or cell lines, suggesting that lncRNAs play an important role on the regulation of adipogenesis and adipogenic differentiation, but few studies have been done in humans.

In the present study, we analyzed lncRNA expression profiles before and after adipogenic differentiation of human adipose-derived mesenchymal stem cells and identified lnc13728 as a novel lncRNA that was significantly upregulated during adipogenic differentiation and specifically expressed in human adipose tissue and its expression was positively correlated with the expression of adipogenic differentiation-related genes in obese individuals. Subsequent experiments suggested that lnc13728 regulated ZBED3 expression levels positively and blocked hADSC adipogenic differentiation by downregulating the WNT/β-catenin pathway. Our findings identify a novel long noncoding RNA, i.e., lnc13728 as a key regulator of adipogenesis, expanding our understanding of the molecular mechanisms in adipocyte differentiation and yielding possible new ideas for intervention in obesity and related disorders.

## Methods

### Isolation and culture of hADSCs

Human adipose tissue was obtained from donors undergoing liposuction. The hADSCs were isolated from the adipose tissue and cultured as previously described [22]. All experiments followed the procedures approved by the Chinese Academy of Medical Sciences and Peking Union Medical College ethics committee.

### Microarray analysis

The hADSCs were exposed to adipogenic differentiation induction medium (AM). Total RNA was extracted using TRIzol (Invitrogen, Waltham, MA, USA) on days 0 and 9. Sample processing and hybridization were conducted by Cnkingbio Biotechnology (Beijing, China) with Affymetrix mRNA and lncRNA microarray chips. Briefly, fold change ≥ 2 (expression value ≥ 3 and *p* < 0.05, day 9 versus day 0) was marked as the cut-off criteria for differentially expressed transcripts.

### Induced adipocyte differentiation and oil red O staining

The hADSCs were exposed to AM, which consisted of high-glucose Dulbecco’s modified Eagle medium (DMEM) containing 10% fetal bovine serum (FBS), 0.1 mM ascorbic acid, 1 μΜ dexamethasone, and 0.5 mM 3-isobutyl-1-methylxanthine. The AM was changed every 3 days until intracellular lipid droplets were observable. Oil red O staining for lipid accumulation was performed as previously described [23].

### RNA isolation and quantitative RT-PCR (qRT-PCR)

Total RNA was extracted from cultured cells with TRIzol (Invitrogen) according to the manufacturer’s instructions. RNA was reverse-transcribed with oligo (dT) primer and M-MLV reverse transcriptase (Takara, Otsu, Japan). qRT-PCR was performed with SYBR Premix Ex Taq (Takara) using QuantStudio 3 (Applied Biosystems, Waltham, USA) in a 10-μL reaction volume. The relative mRNA expression level was evaluated using the comparative threshold cycle (2-ΔΔCt) method and was normalized to the *GAPDH* expression level. Supplementary Table 1 lists the primers used in this study.

### Protein extraction and western blotting

The hADSCs were lysed in radioimmunoprecipitation assay lysis buffer with 1 mM PMSF (Beyotime, Shanghai, China) and protease inhibitor cocktail on ice for 30 min and quantified with a bicinchoninic acid protein assay kit (Beyotime). Protein fractions were separated by 10% sodium dodecyl sulfate–polyacrylamide gel electrophoresis and transferred to polyvinylidene difluoride membranes (0.22 μm, Millipore, Billerica, MA, USA). The membranes were blocked with 5% defatted milk and incubated with specific primary antibodies against PPARγ (1:1000, CST, Beverly, MA, USA), CEBPα (1:1000, CST), AP2 (1:1000, Abcam, Cambridge, UK), CEBPβ (1:1000, CST), ZBED3 (1:1000, GeneTex, Irvine, CA, USA), PLIN1 (1:1000, Abcam), GAPDH (1:10000, Proteintech, Wuhan, China), β-catenin (1:5000, Proteintech), or (p)-GSK3β (1:1000, CST) overnight at 4 °C and then incubated with horseradish peroxidase–conjugated secondary antibody (1:3000, Yeasen, Shanghai, China) at room temperature for 1 h. Immunodetection was visualized using a chemiluminescent ECL reagent (Millipore).

### Cytoplasmic and nuclear RNA extraction

The hADSCs were harvested 3 days after induced adipocyte differentiation. The RNA was extracted according to the manufacturer’s protocol of the NE-PER™ Nuclear and Cytoplasmic Extraction Reagent kit (Thermo Fisher Scientific, Waltham, MA, USA) as previously described [24], and extracted by TRIzol after incubation with proteinase K (10 mg/mL) at 37 °C for 20 min.

### RNA fluorescence in situ hybridization (FISH)

The hADSCs were rinsed briefly in phosphate-buffered saline (PBS), fixed in 4% formaldehyde for 10 min at room temperature, permeabilized in PBS containing 0.5% Triton X-100 (Sigma-Aldrich, St. Louis, MO, USA) for 5 min at 4 °C, then washed in PBS for 3 × 5 min. Hybridization was carried out with a FISH probe (RiboBio, Guangdong, China) at 37 °C in the dark overnight according to the manufacturer’s protocol. Following the RNA FISH, all images were obtained with a confocal microscope.

### Immunofluorescence

The hADSCs were fixed in 4% formaldehyde for 15 min, then permeabilized with 0.2% Triton X-100 for 3 min. PBS with 5% bovine serum albumin (BSA) was then added to block for 1 h at room temperature. Then, the cells were incubated with the primary antibody overnight at 4 °C. After gently washing three times with PBS, the cells were incubated with secondary antibody for 1 h at room temperature and washed with PBS; the chamber containing the cells was dried and mounted with 4′, 6-diamidino-2-phenylindole (DAPI, Invitrogen). Images were recorded by a confocal microscope.

### Antisense oligonucleotide (ASO) transfection and lentiviral vector infection

The cells were transfected with 50 nM lncRNA ASOs or the negative control (NC) (GeneBio, Nanjing, China) using Lipofectamine 3000 (Invitrogen) and Opti-MEM (Gibco, Waltham, MA, USA) culture medium according to the manufacturer’s instructions.

### Statistical analysis

Data were analyzed using GraphPad Prism7 (GraphPad Software Inc., La Jolla, CA, USA). Student’s *t*-tests and one-way analysis of variance (ANOVA) were used to analyze between two groups or among multiple groups, respectively. *p* < 0.05 was considered statistically significant (*p < 0.05, **p < 0.01, ***p < 0.001).

## Results

### Lnc13728 is highly expressed during hADSC adipogenic differentiation

**Fig. 1:**
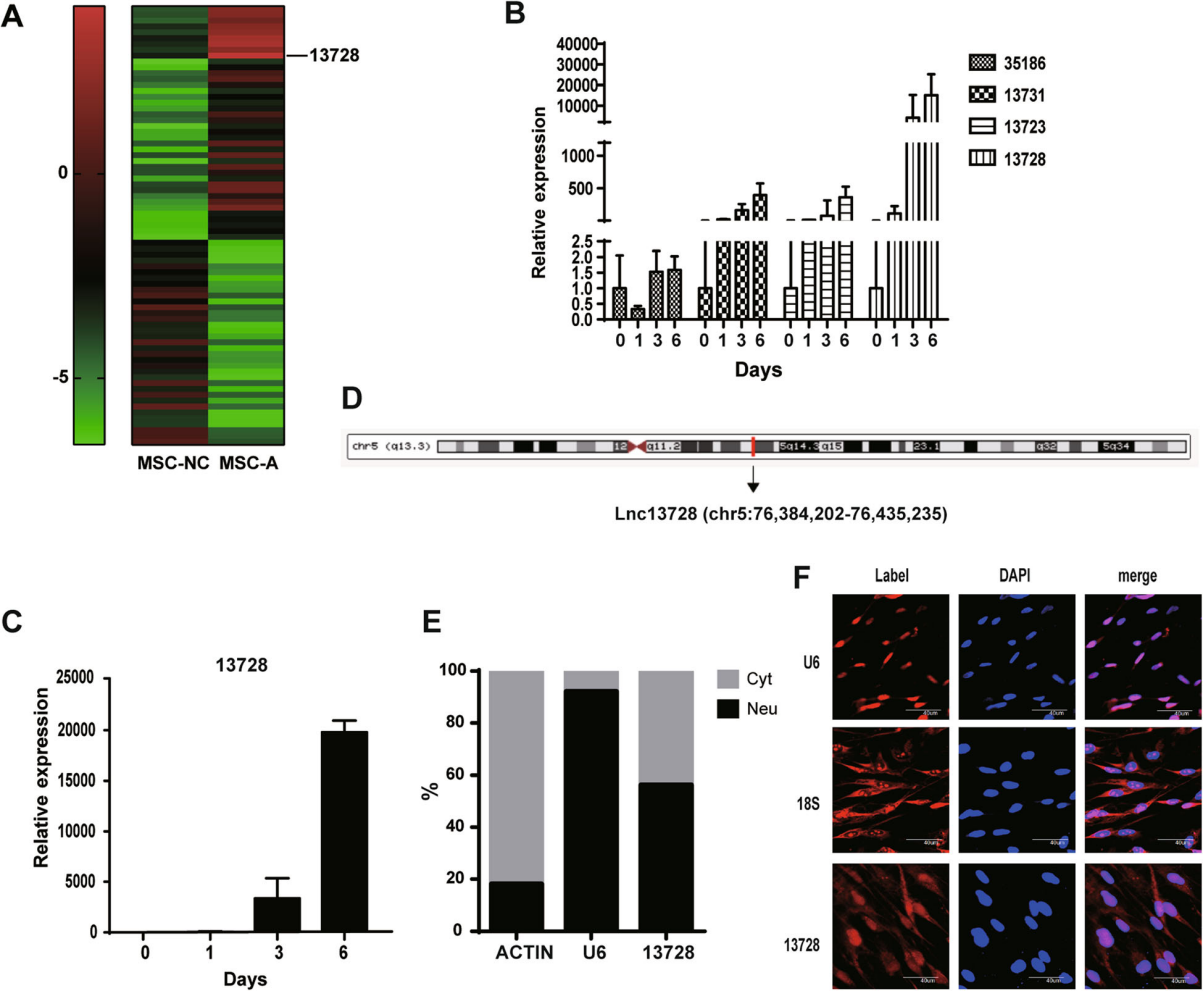
Lnc13728 expression pattern and characterization. **a** Heatmap of upregulated and downregulated lncRNAs of normal and adipogenesis-induced hADSCs. **b** Several lncRNAs were upregulated overtime during hADSC adipogenic differentiation. Their names were based on the lncRNA array probes. **c** qRT-PCR detection of lnc13728 expression during 0–6 days of hADSC adipogenic differentiation. **d** Genome information according to the UCSC Genome Browser showing the location of lnc13728 on the chromosome. **e** qRT-PCR detection of lnc13728 expression levels in hADSC nuclear and cytoplasmic extracts. The cytoplasmic mRNA control was actin, and the nuclear RNA control was U6. **f** RNA FISH detection of lnc13728 subcellular localization in hADSCs. Scale bar 40 μm

We isolated hADSCs from waste adipose tissue obtained after human liposuction and induced their differentiation into adipocytes. The changes in lncRNA expression patterns among adipogenic-induced cells and undifferentiated cells were evaluated via lncRNA arrays, and a heatmap was produced with the data of 40 upregulated and 35 downregulated lncRNAs (Fig. 1a). We identified several lncRNAs that were upregulated during adipogenesis, which was confirmed by qRT-PCR. Among the upregulated lncRNAs, the most significant change was for lnc13728, which was named temporarily after its probe number (Fig. 1b). Lnc13728 expression increased significantly with time during hADSC adipogenic differentiation (Fig. 1c). The basic genome information revealed that lnc13728 was annotated as a 0.54-kb long lncRNA and mapped to chromosome 5 according to the UCSC Genome Browser (http://genome.ucsc.edu/) (Fig. 1d). We performed qRT-PCR and RNA FISH to verify the ratio of lnc13728 expression in the cytoplasm and the nucleus, which indicated that lnc13728 was diffusely distributed throughout the cell (Fig. 1e, f).

### The expression of Lnc13728 is positively correlated with that of adipogenic differentiation-related gene in obese individuals

**Fig. 2:**
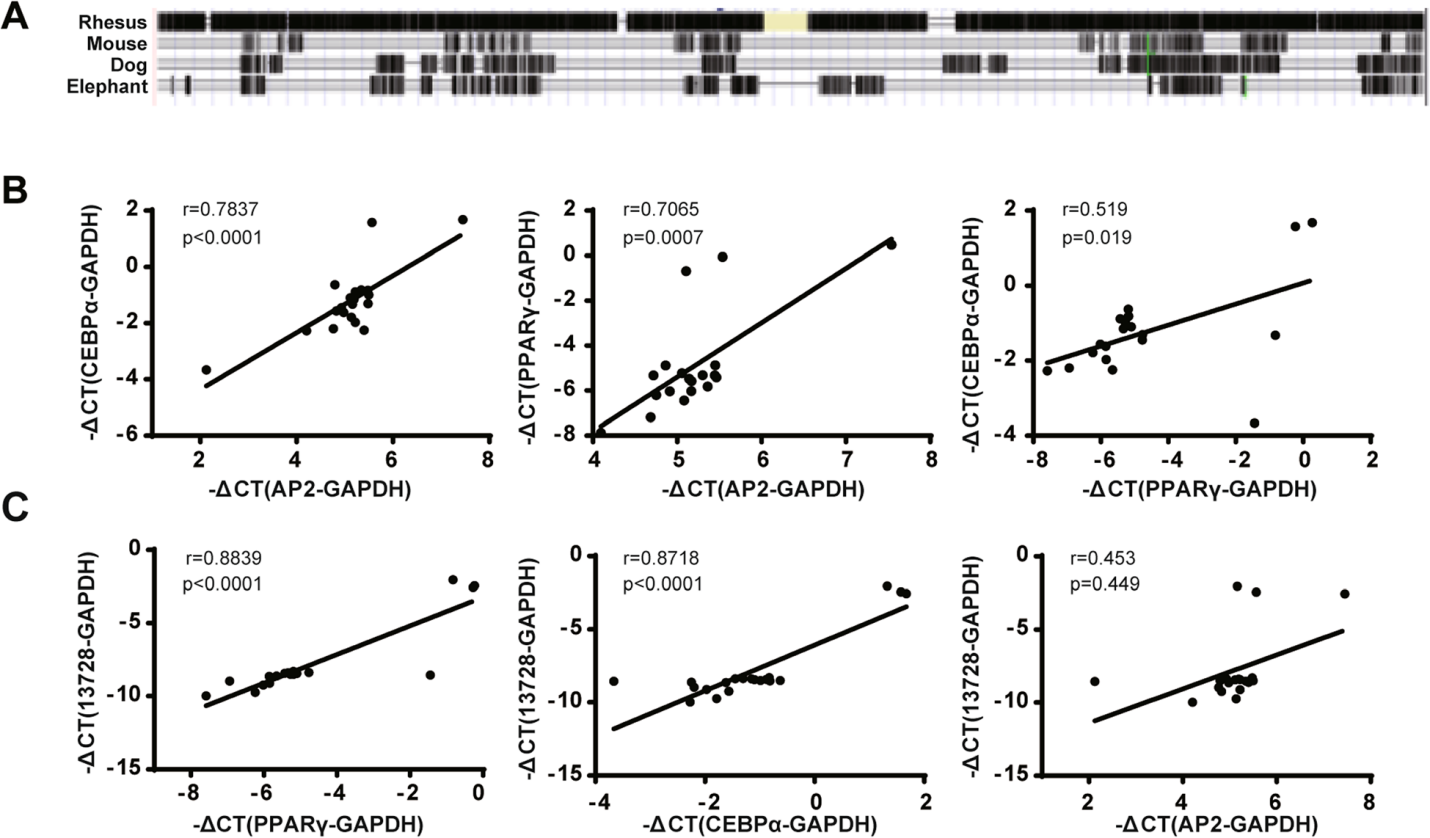
Lnc13728 is not conserved among species and correlated with adipose generation. **a** Conservation analysis of lnc13728 (UCSC Genome Browser). **b**, **c** The correlation between the expression of adipogenesis markers and lnc13728 was analyzed in 20 adipose samples from obese individuals. The internal control was GAPDH

Further interspecies conservative analysis in the UCSC Genome Browser revealed that lnc13728 is only conserved in humans and rhesus monkeys (Fig. 2a). Therefore, we attempted to determine whether it is related to fat production in human tissues. We obtained 20 liposuction adipose tissue samples from obese patients, extracted the total RNA, and used qRT-PCR to analyze the correlation between lnc13728 and the marker genes associated with adipogenic differentiation and adipose formation, such as *PPARγ*, *CEBPα*, and *AP2* (Fig. 2b, c). These data suggest that lnc13728 correlates positively with adipogenesis and may contribute to fat production.

### Knockdown of lnc13728 attenuates hADSC adipogenic differentiation

**Fig. 3:**
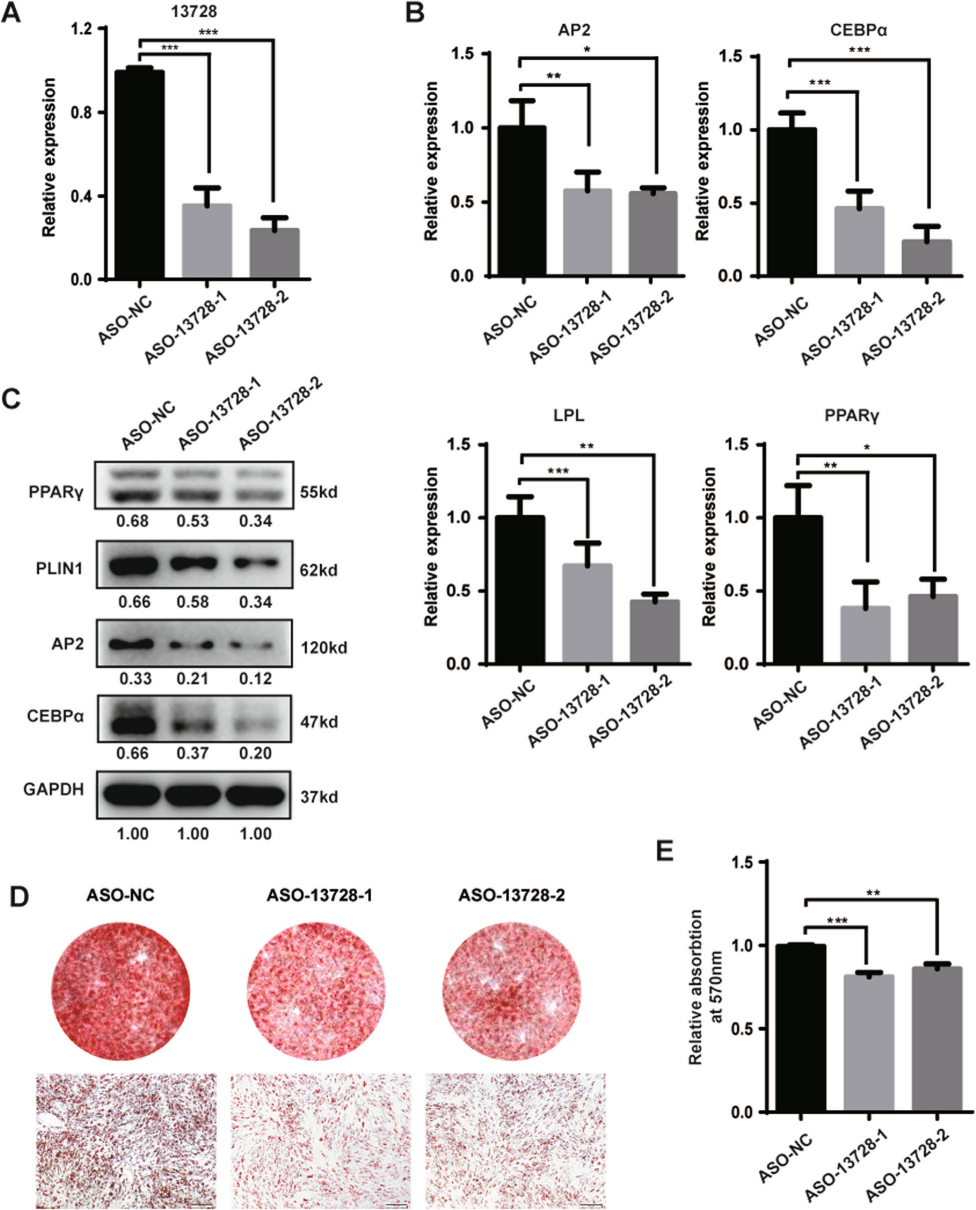
Downregulation of lnc13728 inhibited hADSC adipogenic differentiation. **a** Lnc13728 was silenced in hADSCs via two independent ASOs (ASO-13728-1 and ASO-13728-2). The knockdown efficiency as compared with the NC was verified by qRT-PCR. **b**, **c** qRT-PCR and western blot analyses of the expression of adipogenesis marker genes in hADSCs after knockdown of lnc13728. **d** Oil red O detection of lipid droplet formation. **e** Oil red O was extracted from positive-staining cells by isopropanol, and the absorbance was quantified at 570-nm wavelength. The internal control was GAPDH. Data are the means ± SD (*n* = 3). *p < 0.05; **p < 0.01; ***p < 0.001 compared with the control. Scale bars 200 μm

To validate the function of lnc13728 during adipogenesis, we transfected hADSCs with a set of ASOs and then exposed them to AM (Fig. 3a). qRT-PCR revealed that the intracellular mRNA levels of adipogenic transcription factors and marker genes such as *PPARγ*, *CEBPα*, and *AP2* were downregulated (Fig. 3b). Western blotting showed the same tendency for the corresponding protein expression (Fig. 3c). Consistently, we also found adipogenesis delay due to the knockdown of lnc13728, as shown by the oil red O staining (Fig. 3d) and isopropanol extraction quantitative analysis after staining, to dye and quantity the lipid droplet generation (Fig. 3e). These results suggest that as a promoter, lnc13728 plays an important role in hADSC adipogenesis.

### Lnc13728 overexpression promotes hADSC adipogenic differentiation

**Fig. 4:**
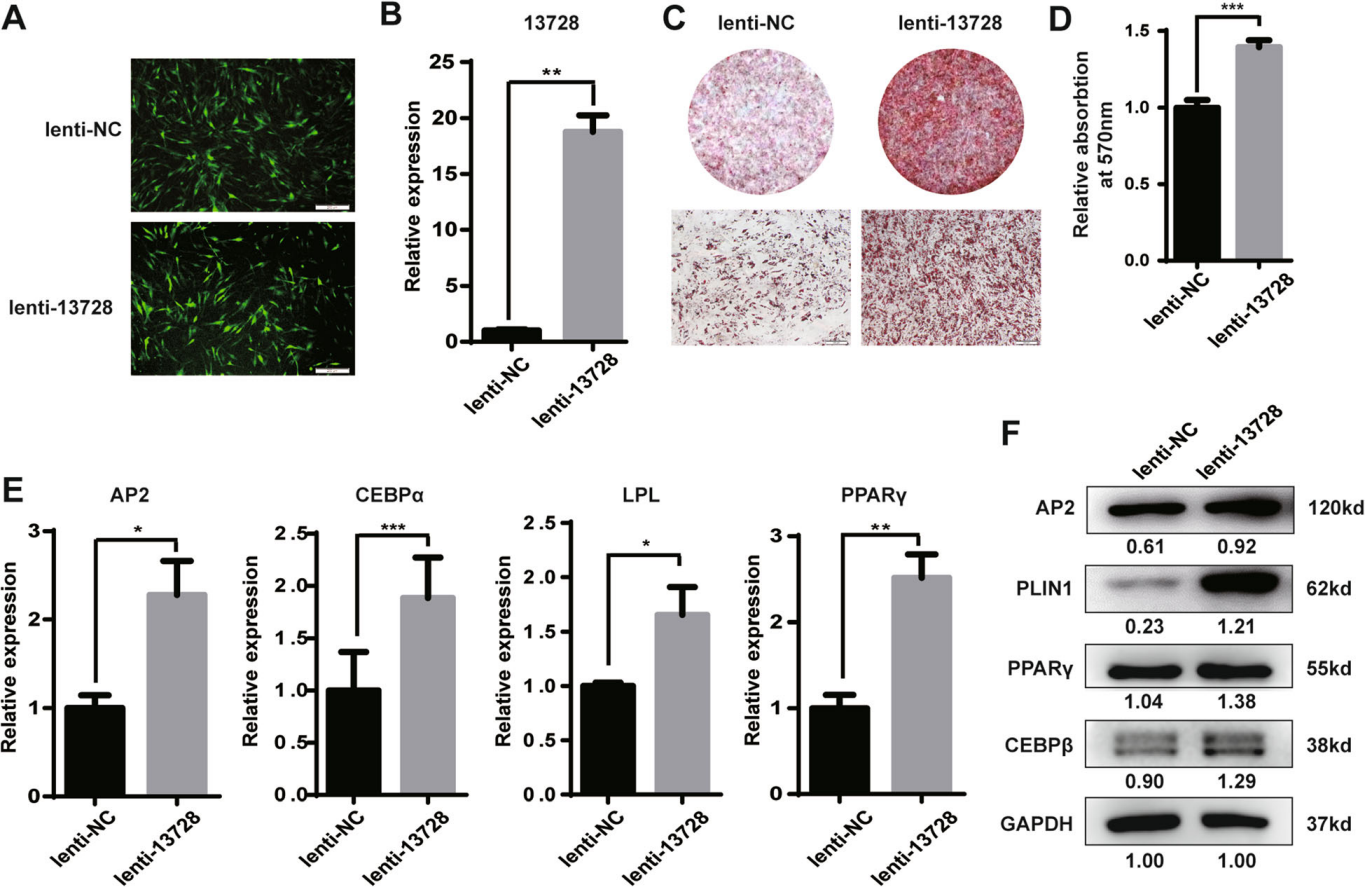
Overexpression of lnc13728 promoted hADSC adipogenic differentiation. **a** Lnc13728 overexpression lentiviral vectors (lenti-13728) and control (lenti-NC) were transfected into hADSCs and indicated by GFP-positive cells. **b** qRT-PCR verification of the overexpression efficiency of lnc13728. **c** Oil red O detection of lipid droplet formation. **d** Oil red O was extracted from positive-staining cells by isopropanol, and the absorbance was quantified at 570-nm wavelength. **e**, **f** qRT-PCR and western blot analyses of the expression of adipogenesis marker genes in hADSCs after upregulation of lnc13728. The internal control was GAPDH. Data are the means ± SD (*n* = 3). *p < 0.05; **p < 0.01; ***p < 0.001 compared with the control. Scale bars 200 μm

To confirm lnc13728 function in hADSC adipogenic differentiation, we infected hADSCs with green fluorescent protein (GFP)-tagged lentivirus vector of lnc13728 (lenti-13,728) or NC (lenti-NC) and purified them with puromycin (Fig. 4a); qRT-PCR analysis indicated that lnc13728 expression was significantly upregulated during adipogenesis (Fig. 4b), and compared with the control, there was enhanced lipid droplet generation indicated by oil red O staining, with the corresponding quantitative analysis of isopropanol extraction (Fig. 4c, d). qRT-PCR and western blotting indicated higher levels of adipogenic marker gene and protein expression, respectively (Fig. 4e, f). Collectively, these data demonstrate that lnc13728 facilitates hADSC differentiation into adipocytes.

### ZBED3 facilitates hADSC adipogenic differentiation

**Fig. 5:**
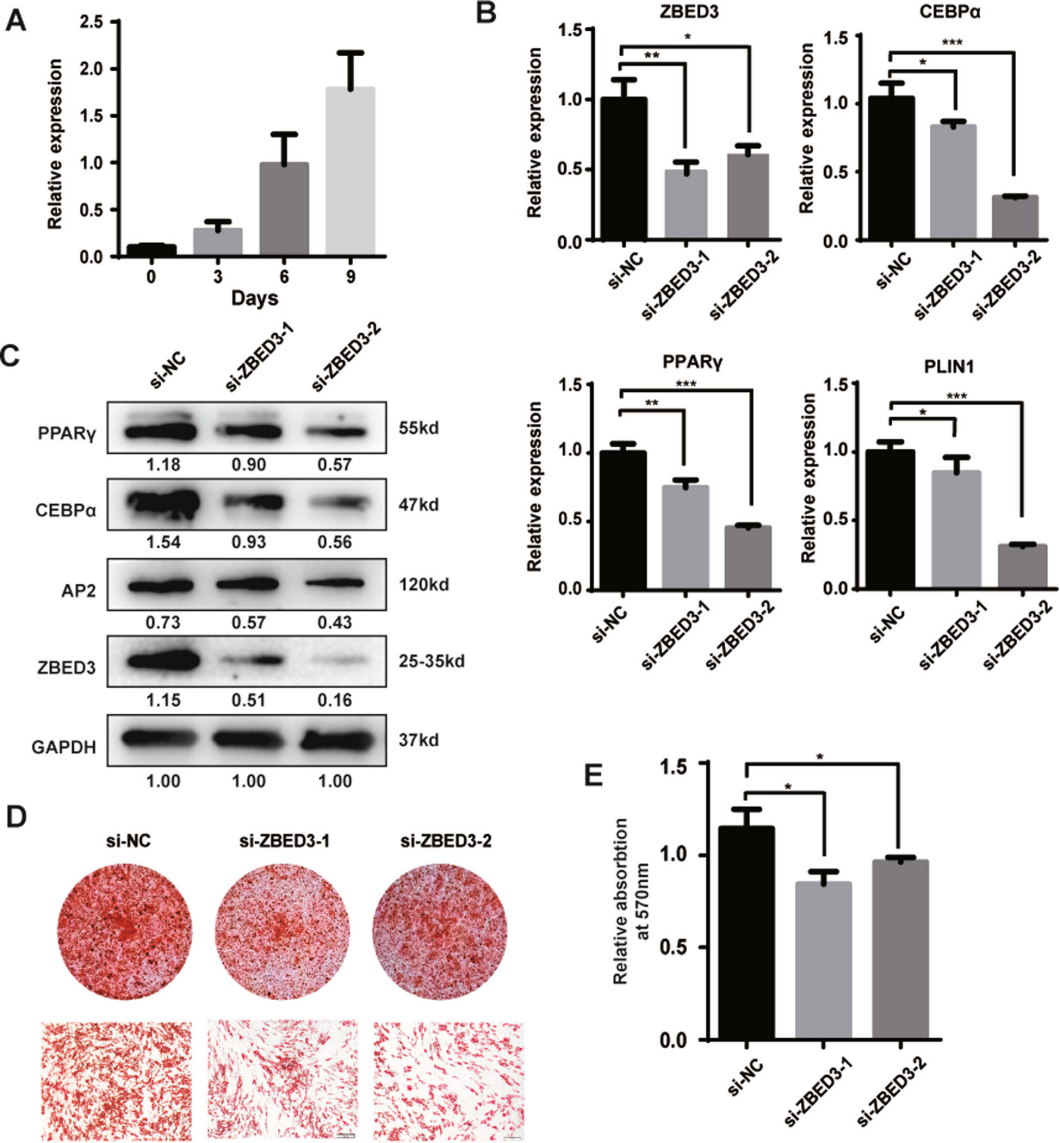
*ZBED3* knockdown inhibited hADSC adipogenic differentiation. **a** qRT-PCR detection of *ZBED3* expression during hADSC adipogenic differentiation. **b**, **c** qRT-PCR and western blot analyses of the expression of ZBED3 and adipogenesis marker genes in hADSCs after knockdown of *ZBED3* via two independent siRNAs (si-ZBED3-1 and si-ZBED3-2). **d** Oil red O detection of lipid droplet formation. **e** Oil red O was extracted from positive-staining cells by isopropanol, and the absorbance was quantified at 570-nm wavelength. The internal control was GAPDH. Data are the means ± SD (*n* = 3). *p < 0.05; **p < 0.01; ***p < 0.001 compared with the control. Scale bars 200 μm

After confirming the effect of lnc13728 in promoting adipogenic differentiation, we explored how it performs this function. The preliminary results showed that lnc13728 is an ncRNA on chromosome 5 (Fig. 1d). Analysis and screening of proteins near its genome sequence showed a coding protein near where lnc13728 is located, i.e., ZBED3, that caught our attention. qRT-PCR revealed that *ZBED3* expression increased gradually during hADSC adipogenic differentiation (Fig. 5a). Based on the expression profile, we inferred that ZBED3 and lnc13728 may play a similar role during hADSC adipogenic differentiation. To verify it, we knocked down *ZBED3* with small interfering RNA (siRNA) in hADSCs and induced them into adipogenic lineages. qRT-PCR and western blotting respectively showed downregulated adipogenesis marker gene and protein expression levels after *ZBED3* inhibition (Fig. 5b, c). Dyeing and quantity of lipid droplets by oil red O staining and quantitative analysis of isopropanol extraction revealed that the lipid droplet generation of cells was decreased by *ZBED3* downregulation (Fig. 5d, e). Therefore, these findings demonstrate that ZBED3 plays a positive regulatory role in hADSC adipogenic differentiation.

### Lnc13728 regulates ZBED3 expression positively and downregulates the WNT/β-catenin pathway

**Fig. 6:**
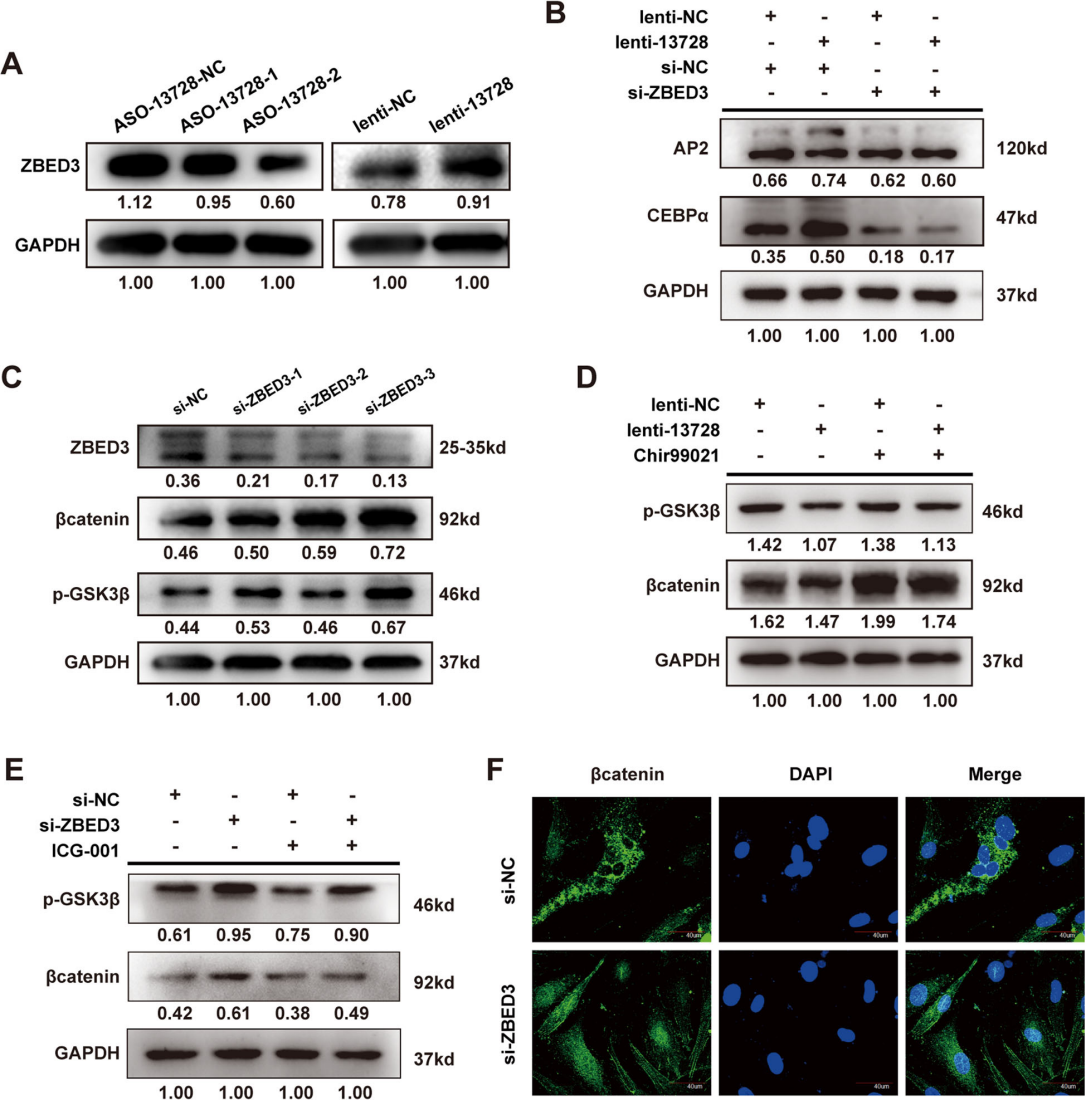
Lnc13728 regulated ZBED3 expression positively, thereby inhibiting the WNT/β-catenin pathway. **a** Western blot detection of ZBED3 expression after lnc13728 knockdown or overexpression. **b** Western blot detection of adipogenesis-related proteins after lnc13728 overexpression and *ZBED3* knockdown. **c** hADSCs were treated with Chir99021 (3 μM) after lnc13728 overexpression and expression of WNT-related proteins (β-catenin and p-GSK3β) was detected by western blot. **d** Western blot detection of β-catenin and p-GSK3β in *ZBED3* knockdown hADSCs or control hADSCs. **e** p-GSK3β and β-catenin were analyzed by western blot in hADSCs undergoing adipogenic differentiation after ZBED3 knockdown and ICG001 (20 μM) treatment. **f** Immunofluorescence detection of subcellular localization of β-catenin. Scale bar 40 μm. The internal control was GAPDH

We performed western blotting and qRT-PCR to reveal the relationship between lnc13728 and ZBED3, and how hADSC adipogenesis is promoted by lnc13728. We observed decreased ZBED3 expression in the hADSCs with lnc13728 knockdown, the opposite was true for the lnc13728 overexpression group, but there was no significant change in lnc13728 expression after *ZBED3* knockdown (Fig. 6a, Supplementary Figure 1a). These results suggested that lnc13728 may be a post-transcriptional regulator of ZBED3. LncPro, a bioinformatics tool, predicted that ZBED3 may interact with lnc13728, and yielded a score of > 50, which means that a pair may interactive [25] (Supplementary Figure 1b). Western blot results showed that in the case of ZBED3 knockdown, adipogenic differentiation of hADSC was not affected by overexpression of lnc13728 (Fig. 6b), suggesting that lnc13728 is dependent on ZBED3 to regulate adipogenic differentiation.

On the other hand, after downregulation of ZBED3, the expression of proteins related to the WNT/β-catenin pathway, such as β-catenin and phosphorylated (p)-GSK3β, were increased (Fig. 6c). The WNT activator Chir99021 and inhibitor ICG001 offset the effects of overexpression of lnc13728 and knockdown of ZBED3 on the WNT/β-catenin pathway in hADSC adipogenic differentiation respectively (Fig. 6d, e). Immunofluorescence showed that more β-catenin entered the nucleus after *ZBED3* knockdown, which suggested the activation of the WNT signaling pathway (Fig. 6f). Collectively, our results suggested that lnc13728 promotes hADSC adipogenic differentiation possibly by regulating the expression of ZBED3 which plays a role in inhibiting the WNT/β-catenin pathway.

## Discussion

The risks of obesity are well known, and there are no effective clinical treatments for now. Plenty of regulatory factors involved in the progression of obesity. Here we focused on long noncoding RNAs, which have high tissue specificity and could be diagnosis biomarkers for diseases [26, 27]. More and more studies have revealed that lncRNAs have a wide range of regulatory functions in obesity-related adipogenesis and lipid metabolism [28, 29]. On the one hand, lncRNA that plays a key regulatory role in adipogenic differentiation can be used as a diagnostic marker for obesity prevention as most of the previous application cases [30–32]; on the other hand, although the effect of inhibiting MSC adipogenic differentiation on existing obesity remains to be clarified, whether the ASO [33, 34] targeting lncRNA can be developed and utilized to intervene in obesity or at least prevent more serious obesity is still worth further study. However, most current researches based on mouse-derived cells or cell lines, but lncRNAs have poor conservation across species, which appears to be an inescapable barrier for comprehensively understanding the development of human adipose and future clinical application. Thus, we need to concentrate more on human tissue specifically expressed lncRNA to exert practical value for intervention of obesity and adipogenesis-related disease. Our team members have previously reported that the lncRNA *ADINR* binds to PA1 on the histone methyltransferase complex MLL3/4, which is recruited to the promoter region of the adipogenic core transcription factor CEBPα, and activates CEBPα transcription by promoting K3K4me3 methylation, facilitating human adipose-derived MSC (hADSC) adipose production [35]. In this study, we identified the high abundance of lnc13728 in the adipocyte induction process of hADSCs through high-throughput lncRNA expression profiling and systematically studied how lnc13728 regulates hADSC adipogenesis and adipogenic differentiation.

Lnc13728 is poorly conserved, and human-specific lnc13728 expression has much practical significance for clinical application. The background level of lnc13728 in hADSCs was low, but that its expression increased sharply during adipogenesis, suggesting its unique role in this process. Considering the positive correlation between the expression of adipogenic transcription factors and lnc13728 in the discarded adipose samples from obese patients undergoing liposuction, we conducted an in-depth study on the role of lnc13728 in adipogenesis and validated that lnc13728 has a positive impact on this process.

LncRNAs function in diverse ways [36–38]. To further explore how lnc13728 promotes adipogenic differentiation, we analyzed the location of lnc13728 on the genome and neighboring genes and investigated related proteins. We identified *ZBED3* on the upstream antisense strand of lnc13728 on chromosome 5. We found that lnc13728 can regulate the expression of *ZBED3* at the post-transcriptional level, while *ZBED3* has no effect on the expression of lnc13728. It was reported that *ZBED3* mRNA and protein in muscle and fat were significantly increased in both db/db mice and T2DM patients and associated with insulin resistance [39]. Obesity is one of the important causes of diabetes, so we studied the role of ZBED3 in adipogenesis and found that *ZBED3* expression increased gradually during hADSC adipogenic differentiation and have positive impact on this process, which was consistent with lnc13728.

Next, we continue to investigate the possible mechanism through which ZBED3 functions. Current studies showed that ZBED3 is related to the WNT/β-catenin pathway in tumor cells and that promotes cancer proliferation by regulating β-catenin expression by interacting with Axin [40]. The WNT/β-catenin pathway controls cell proliferation, development, and fate determination, disorders of which are related to a series of human diseases, such as tumors, osteoporosis, and obesity [41–44]. Generally, the WNT/β-catenin pathway has a crucial effect on adipose generation through inhibitory regulation [41, 45]. WNT/β-catenin signaling maintains preadipocyte undifferentiated status by inhibiting the production of the adipogenesis transcription factors C/EBPα and PPARγ [13, 45]. Disorder of the intracellular or extracellular WNT/β-catenin pathway through constitutive expression of Axin results in spontaneous adipocyte differentiation [45]. Many molecules regulate the WNT/β-catenin pathway during adipogenic differentiation, but there are few reports on the regulatory role of lncRNA in this process. We explored that ZBED3 negatively regulated WNT/β-catenin pathway during hADSC adipogenic differentiation, which appears inconsistent with its function in previous research on tumor cells. We hypothesized that the specifically elevated lnc13728 in hADSC may block the role of ZBED3 and WNT-related proteins and enables ZBED3 to perform different functions in adipocyte differentiation. Our study still has limitations; the precise mechanisms by which ZBED3 affects the WNT/β-catenin pathway in hADSCs and how lnc13728 interacts with ZBED3 remain to be ascertained.

## Conclusion

We first identified lnc13728 and confirmed its function as a novel positive regulator during human adipogenic differentiation. Lnc13728 is specifically and highly expressed during hADSC adipogenesis and facilitates this process possibly by positively regulating the levels of ZBED3 and subsequently downregulating the WNT/β-catenin pathway. Our findings provide new evidence of the intermolecular mechanisms of adipocyte development and new insights to the regulatory role of human-specific lncRNAs in obesity.

## Supplementary Information

**Additional file 1: Supplementary Table 1.** Sequence of primers used in the study.

**Additional file 2: Supplementary Figure 1. a** qRT-PCR detection of lnc13728 expression after *ZBED3* knockdown. **b** The interaction between lnc13728 and ZBED3 was predicted by LncPro, which considered a value of 50 as the cut-off. Data are the means ± SD (*n* = 3). *p < 0.05; **p < 0.01; ***p < 0.001 compared with the control.

## Data Availability

All data generated or analyzed during this study are included in this published article.

## Abbreviations

MSCs: Mesenchymal stem cells
hADSCs: Human adipose-derived mesenchymal stem cells
LncRNAs: Long noncoding RNAs
Lnc13728: Long noncoding RNA 13728
PPARγ: Peroxisome proliferator-activated receptor γ
CEBPα: CCAAT enhancer binding proteinα
CEBPβ: CCAAT enhancer binding proteinβ
AP2: Adipocyte protein 2
LPL: Lipoprotein lipase
PLIN1: Perilipin-1
ZBED3: Zinc finger BED domain-containing protein 3
p-GSK3β: Phosphorylated glycogen synthase kinase-3 β
ASOs: Antisense oligonucleotides
GAPDH: Glyceraldehyde-3-phosphate dehydrogenase
DAPI: 4′,6-Diamidino-2-phenylindole
qRT-PCR: Quantitative real-time polymerase chain reaction
FISH: Fluorescence in situ hybridization
NC: Negative control
