# Supplementary material for: Lnc13728 facilitates human mesenchymal stem cell adipogenic differentiation via positive regulation of ZBED3 and downregulation of the WNT/β-catenin pathway
Source: Stem Cell Res Ther. 2021 Mar 12;12:176. doi: 10.1186/s13287-021-02250-8 (PMC7953623; doi:10.1186/s13287-021-02250-8)
Supplement: Supplementary file 1 — Additional file 1: Supplementary Table 1. Sequence of primers used in the study. [file 13287_2021_2250_MOESM1_ESM.docx]

**Supplementary Table 1** Sequence of primers used in the study

| **Gene** | **Primer sequences** (5′–3′) |
| --- | --- |
| **lnc13728** | Forward (F): GTCACACATGGACTTGAAGGA |
|  | Reverse (R): GCAATCCCACTCCAGGTATTTA |
| **ZBED3** | F: TTCCGTCCGGGACAAAATGT  AACCATCAGCAGTGCCCTTT |
|  | R: AACCATCAGCAGTGCCCTTT |
| **PPARγ** | F: TGAACGTGAAGCCCATCGAG  R: CTTGGCGAACAGCTGAGAGG |
|  | R: CTTGGCGAACAGCTGAGAGG |
| **CEBPα** | F: GCGCAAGAGCCGAGATAAAG  R: CGGTCATTGTCACTGGTCAACT |
|  | R: CGGTCATTGTCACTGGTCAACT |
| **AP2** | F: ACTGGGCCAGGAATTTGACG  R: CTCGTGGAAGTGACGCCTT  R: CTCGTGGAAGTGACGCCTT |
|  | R: CTCGTGGAAGTGACGCCTT |
| **LPL** | F: TCATTCCCGGAGTAGCAGAGT  R: GGCCACAAGTTTTGGCACC |
|  | R: GGCCACAAGTTTTGGCACC |
| **PLIN1** | F: CCATGTCCCTATCAGATGCCC |
|  | R: CTGGTGGGTTGTCGATGTC |
| **Actin** | F: CATGTACGTTGCTATCCAGGC |
|  | R: CTCCTTAATGTCACGCACGAT |
| **U6** | F: AATCTAGCTGCTGCGGTTC |
|  | R: GAGGATTCGCTGACGGTTAAA |
| **GAPDH** | F: GGTCACCAGGGCTGCTTTTA |
|  | R: GGATCTCGCTCCTGGAAGATG |
