# Supplementary figures and images for: Lnc13728 facilitates human mesenchymal stem cell adipogenic differentiation via positive regulation of ZBED3 and downregulation of the WNT/β-catenin pathway
Source: Stem Cell Res Ther. 2021 Mar 12;12:176. doi: 10.1186/s13287-021-02250-8 (PMC7953623; doi:10.1186/s13287-021-02250-8)

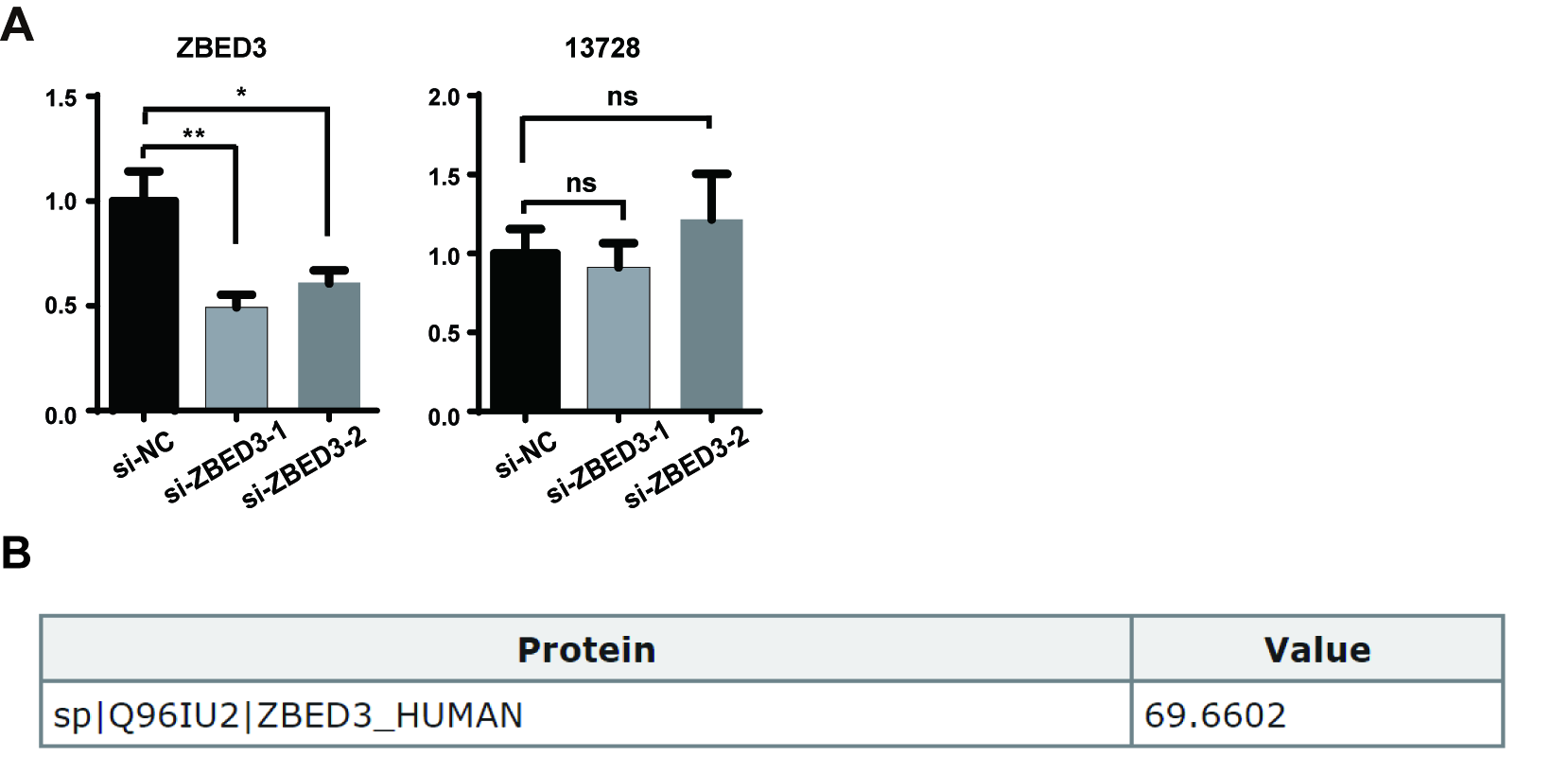

Supplement: Supplementary file 2 — Additional file 2: Supplementary Figure 1. a qRT-PCR detection of lnc13728 expression after ZBED3 knockdown. b The interaction between lnc13728 and ZBED3 was predicted by LncPro, which considered a value of 50 as the cut-off. Data are the means ± SD (n = 3). *p < 0.05; **p < 0.01; ***p < 0.001 compared with the control. [file 13287_2021_2250_MOESM2_ESM.tif]
